# Curcumin Alleviates the Senescence of Canine Bone Marrow Mesenchymal Stem Cells during In Vitro Expansion by Activating the Autophagy Pathway

**DOI:** 10.3390/ijms222111356

**Published:** 2021-10-21

**Authors:** Jiaqiang Deng, Ping Ouyang, Weiyao Li, Lijun Zhong, Congwei Gu, Liuhong Shen, Suizhong Cao, Lizi Yin, Zhihua Ren, Zhicai Zuo, Junliang Deng, Qigui Yan, Shumin Yu

**Affiliations:** 1 College of Veterinary Medicine, Sichuan Agricultural University, Chengdu 611130, China; 18408205642@163.com (J.D.); Ouyang.ping@live.cn (P.O.); liweiyao2021@126.com (W.L.); 18728571971@163.com (L.Z.); gcw081543@swmu.edu.cn (C.G.); shenlh@sicau.edu.cn (L.S.); suizhongcao@126.com (S.C.); yinlizi@sicau.edu.cn (L.Y.); zhihua_ren@126.com (Z.R.); ZZCJL@126.com (Z.Z.); dengjl213@126.com (J.D.); 2 College of Life Sciences, Sichuan University, Chengdu 610064, China; 3 Laboratory Animal Centre, Southwest Medical University, Luzhou 646000, China

**Keywords:** curcumin, senescence, autophagy, canine bone marrow-derived mesenchymal stem cells

## Abstract

Senescence in mesenchymal stem cells (MSCs) not only hinders the application of MSCs in regenerative medicine but is also closely correlated with biological aging and the development of degenerative diseases. In this study, we investigated the anti-aging effects of curcumin (Cur) on canine bone marrow-derived MSCs (cBMSCs), and further elucidated the potential mechanism of action based on the modulation of autophagy. cBMSCs were expanded in vitro with standard procedures to construct a cell model of premature senescence. Our evidence indicates that compared with the third passage of cBMSCs, many typical senescence-associated phenotypes were observed in the sixth passage of cBMSCs. Cur treatment can improve cBMSC survival and retard cBMSC senescence according to observations that Cur (1 μM) treatment can improve the colony-forming unit-fibroblasts (CFU-Fs) efficiency and upregulated the mRNA expression of pluripotent transcription factors (SOX-2 and Nanog), as well as inhibiting the senescence-associated beta-galactosidase (SA-β-gal) activities and mRNA expression of the senescence-related markers (p16 and p21) and pro-inflammatory molecules (tumor necrosis factor-α (*TNF-α*) and interleukin-6 (IL-6)). Furthermore, Cur (0.1 μM~10 μM) was observed to increase autophagic activity, as identified by upregulation of microtubule-associated protein 1 light chain 3 (LC3), unc51-like autophagy-activating kinase-1 (ULK1), autophagy-related gene (Atg) *7* and Atg12, and the generation of type II of light chain 3 (LC3-II), thereby increasing autophagic vacuoles and acidic vesicular organelles, as well as causing a significant decrease in the p62 protein level. Moreover, the autophagy activator rapamycin (RAP) and Cur were found to partially ameliorate the senescent features of cBMSCs, while the autophagy inhibitor 3-methyladenine (3-MA) was shown to aggravate cBMSCs senescence and Cur treatment was able to restore the suppressed autophagy and counteract 3-MA-induced cBMSC senescence. Hence, our study highlights the important role of Cur-induced autophagy and its effects for ameliorating cBMSC senescence and provides new insight for delaying senescence and improving the therapeutic potential of MSCs.

## 1. Introduction

Aging and longevity have always been seductive topics for organisms. As a type of adult stem cells with the capacity for self-renewal, immunosuppression, differentiation, and migration, mesenchymal stem cells (MSCs) extensively reside within various tissues and organs, such as bone marrow, adipose tissue, amniotic fluid, placenta, umbilical cord, and muscle [1,2], and play an essential role in maintaining tissue homeostasis throughout the lifespan of an organism [3,4]. Accumulating evidence has revealed that biological aging and the development of many degenerative diseases can attribute to MSC senescence [5,6]. In addition, MSCs are promising sources of cell-based regenerative therapy, but usually need to be expanded in vitro to achieve the minimum transplantation number of MSCs (20–100 million) for treatments [7]. However, in vitro culture is not able to completely simulate the in vivo microenvironment of MSCs, and inevitably triggers cellular senescence [8,9]. Senescence is mitotic cells occurs in response to a variety of stressors, such as telomere dysfunction [10], DNA damage [11], oxidative stress [12], and oncogene activation [13], and ultimately leads to the termination of cell division. Moreover, senescent MSCs exhibit a negative effect on biological functions, including immunomodulation, differentiation, and migration [14]. Much work has attempted to establish effective strategies to retard senescence in MSCs. Recently, several herb-derived products have been suggested to promote organism health and longevity and may be promising tools for delaying MSC senescence [15].

Curcumin (Cur), a hydrophobic polyphenol extracted from traditional Chinese medicine turmeric, has attracted great attention due to its anti-inflammatory, antioxidant, anti-apoptotic, anti-tumor, and anti-aging effects in tissue cells and some disease models [16,17,18,19]. Numerous studies have demonstrated that Cur has health-related benefits in age-related diseases, including osteoarticular, neurological, reproductive, and cardiovascular diseases [20,21,22,23,24]. Aging is associated with musculoskeletal changes and the initiation and progression of osteoarticular diseases. Chronic dietary intervention with Cur was found to ameliorate the motor function of the hand and digits in middle-aged monkeys and had beneficial effects on aged skeletal muscle [25,26]. Available data from Buhrmann and colleagues have revealed that Cur can attenuate environment-derived osteoarthritis by balancing chondrocyte survival and inflammatory responses [27,28]. Additionally, Cur exerts neuroprotective effects via mediating autophagy and inflammation and may therefore be an effective therapy for patients with neurodegenerative diseases, such as Alexander disease, Alzheimer’s disease, and Parkinson’s disease [29,30].

Indeed, the fact that Cur possesses pleiotropic activity could be attributed to their activation and protection effects on MSCs [31,32]. Several studies have confirmed that Cur is involved in regulating the immunomodulatory capabilities; the differential potential of MSCs to different cell lineages (neurocyte, chondrocyte, adipocyte, and osteoblast), and in multiple signaling mechanisms is involved in this process [28,31,33,34,35]. Moreover, Cur is also regarded as a good antioxidant by which to improve the lifespan of rat adipose tissue-derived mesenchymal stem cells (ADSCs) due to the increase in the telomerase reverse transcriptase (TERT) gene expression [36]. A recent study showed that the inhibition of p65 by Cur can prevent cellular senescence and inflammatory activation in human umbilical cord-derived MSCs [37]. Obviously, more attention has been focused on the anti-aging effects of Cur in MSCs due to their enormous therapeutic potential, but the mechanisms underlying this effect are still unclear. Some evidence from in vitro studies has confirmed that the beneficial effects exerted by Cur should be attributed to its modulation of autophagy [29,38,39,40,41]. Han and colleagues demonstrated that Cur can protect human umbilical vein endothelial cells (HUVECs) from H_2_O_2_-induced oxidative damage, and that positive effects occur by the enhancement of autophagy via phosphatidylinositol 3-kinase (PI3K)/Akt/mammalian target of rapamycin (mTOR) inhibition [40]. Another study showed that Cur is able to promote cell survival by inhibiting hypoxia/reoxygenation (H/R)-induced apoptosis and excessive autophagy among cardiomyocytes [41]. Therefore, whether Cur performs as a positive or negative regulator of autophagy depends on the stress-producing stimulus and cellular setting, and more details are required to determine whether the modulation of autophagy by Cur is an effective strategy for delaying MSC senescence.

As a principle of degradation and recycling pathway for intracellular substances, autophagy plays a crucial role in maintaining cellular homeostasis and withstanding environmental pressure and has exhibited the enormous potential for delaying cellular senescence and treating age-related diseases. Organelle dysfunction and toxic metabolite accumulation are the key characteristic of senescent MSCs [42], thus suggesting a potential link between autophagy and senescence. Ma and colleagues found that the autophagic activity of aged BMSCs was diminished in comparison with young BMSCs and that autophagy inhibitor 3-methyladenine (3-MA) could accelerate senescence in young MSCs; in contrast, they found that autophagy activator rapamycin (RAP) could partially restore the biological properties of aged BMSCs [5]. A recent study examined the role of premodulated autophagy through the employment of RAP and 3-MA in MSC senescence induced by D-galactose (D-gal), and showed that RAP remarkably alleviated MSC senescence [43]. Interestingly, several studies have demonstrated that an increase in autophagy is observed in senescent MSCs and that autophagy is indispensable for maintaining the MSC senescence processes [44,45]. Therefore, whether autophagy could be regarded as a positive or negative regulator of senescence in MSCs remains conflicting and ambiguous. Consequently, it would be interesting to investigate the relationship between autophagy, MSC senescence, and Cur, which could provide a wider perspective for delaying senescence and improving the therapeutic potential of MSCs.

In this study, our primary purpose was to elucidate whether Cur treatment could delay canine bone marrow-derived MSC (cBMSC) senescence and whether the underlying mechanism was correlated with the modulation of autophagy. To this end, we evaluated the phenotypic characterization, autophagic activity, and gene expression after exposure to Cur in senescent cBMSCs, as well as whether the relationship between autophagy and its effects on cBMSCs senescence is determined by using 3-MA and RAP.

## 2. Results

### 2.1. Characteristics of cBMSCs

**Figure 1:**
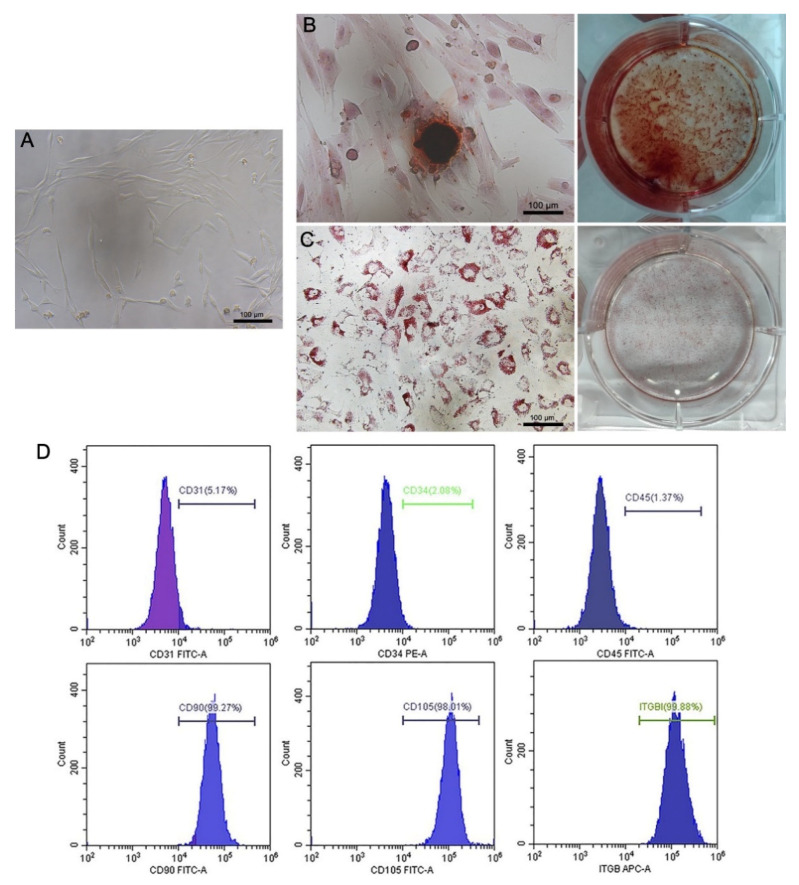
Characteristics of MSCs isolated from canine bone marrow. (**A**) The morphology of third-generation canine bone marrow-derived mesenchymal stem cells (cBMSCs) cultured at 48 h was observed using a light microscope; scale bars = 100 μm. (**B**) Alizarin Red S stained calcium deposition; scale bars = 100 μm. (**C**) Oil Red O staining was used to observe the lipid droplet accumulation; scale bars = 100 μm. (**D**) The 3rd passage cBMSCs were stained with FITC, PE, or APC fluorescent-labeled antibodies and analyzed using flow cytometry.

cBMSCs were expanded in vitro with standard procedures and were shown to be stably passaged over nine generations (data not shown). The cBMSCs displayed a plastic-adherent and fibroblast-like appearance throughout the culturing process (Figure 1A). The differentiation capacity of cBMSCs was confirmed by adipogenic and osteogenic induction (Figure 1B,C). The immunophenotype of cBMSCs was evaluated using flow cytometry. The results showed that the cell population positively expressed CD90, CD105, and ITGB1, and negatively expressed CD31, CD34, and CD45, suggesting their mesenchymal rather than hematopoietic origin (Figure 1D and Table 1).

### 2.2. cBMSCs Progressively Display Senescent Features along Expansion In Vitro

**Figure 2:**
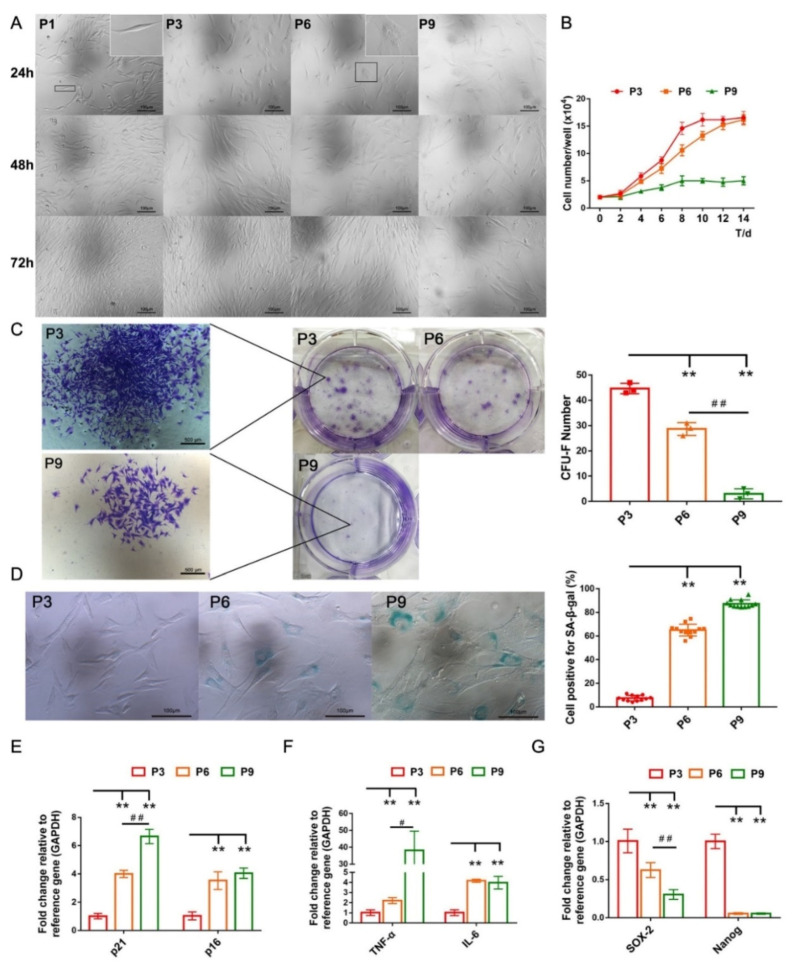
Senescence characteristics of cBMSCs after expansion in vitro. (**A**) Cell morphology of cBMSCs (P1, P3, P6, and P9) cultured in vitro for 24 h, 48 h, and 72 h; scale bars = 100 μm. (**B**) Growth curve of cBMSCs (P3, P6, and P9) was determined by cell counting. The proliferation capacity of cBMSCs was gradually decreased over the process of long-term cultivation and they underwent growth arrest at P9. (**C**) CFU-F assays of cBMSCs at P3, P6, and P9. The number of colonies was estimated after culturing in vitro for 14 d. (**D**) Representative image of SA-β-gal staining and percentage of SA-β-gal positive cells in cBMSCs at P3, P6, and P9. The expression of senescence-related genes of *p21* and *p16* (**E**), pro-inflammatory cytokine genes *TNF-α* and *IL-6* (**F**), and stemness markers *Nanog* and *SOX-2* (**G**) measured by RT-qPCR. Gene expression was normalized relative to the expression of *GAPDH*. These values are the mean ± SD of triplicate experiments. ** *p* < 0.01 compared with P3. ^#^ *p* < 0.05, ^##^ *p* < 0.01 for intragroup comparisons.

Early-passage cBMSCs (P1 and P3) displaying a long fusiform or triangular shape, and arranged into a whirlpool after growth in vitro (Figure 2A). Subsequently, cBMSCs gradually displayed senescence-associated phenotypes over the course of long-term cultivation in vitro, characterized by an enlarged and flat morphology and a reduction in proliferation (Figure 2A,B). Additionally, the results from a colony-forming unit-fibroblast (CFU-F) assay showed that both the number of CFU-F and the size of colonies were significantly diminished along culture passages (Figure 2C), consistent with the downregulated mRNA expression of pluripotent transcription factors *Nanog* and *SOX-2* in the 6th and 9th passage (Figure 2G).

Senescence-associated β-galactosidase (SA-β-gal) is a lysosomal enzyme, the activity of which is strongly correlated with cellular senescence [46]. The results of SA-β-gal staining indicated that the number of SA-β-gal positive cells grew from 7.5 ± 2.4% (P3) to 65.0 ± 5.0% (P6) and 87.3 ± 3.3% (P9), respectively (Figure 2D). Additionally, increases in the expression of cell cycle kinase inhibitors (*p21* and *p16*) and pro-inflammatory molecules (*TNF-α* and *IL-6*), which act as components of a senescence-associated secretory phenotype (SASP), were detected in the 6th and 9th passage (Figure 2E,F). Therefore, cBMSCs exhibit a passage-dependent increase in senescent phenotypes and cease proliferation at the 9th passage.

### 2.3. Cur Alleviates the Senescent State of cBMSCs

**Figure 3:**
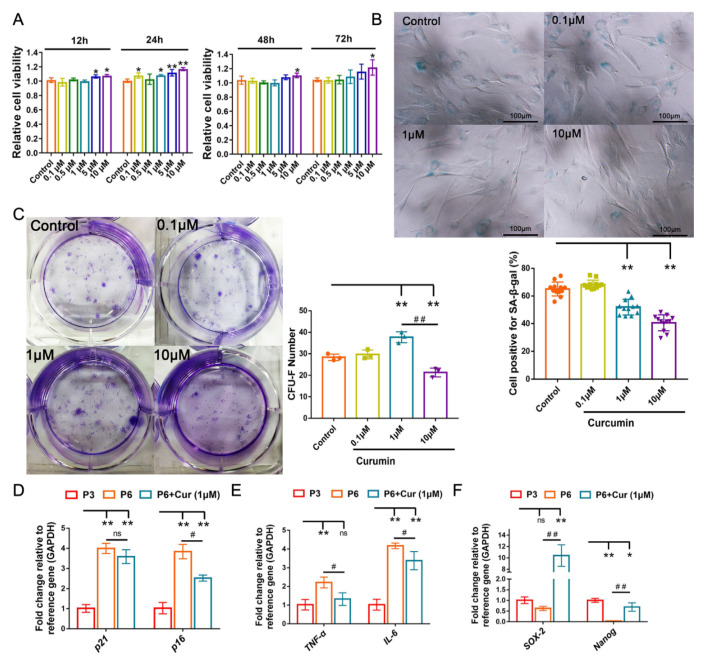
Effects of Cur on senescent cBMSCs (P6). (**A**) Effect of Cur on the viability of cBMSCs. Effects of different doses (0.1, 0.5, 1, 5, and 10 μmol/L) and time points (12 h, 24 h, 48 h, and 72 h) of the responses of Cur on the viability of P6 cBMSCs were evaluated using a CCK-8 assay. (**B**) SA-β-gal expression levels in the P6 cBMSCs in the absence (control) or presence of Cur (0.1, 1, and 10 μmol/L) for 24 h; scale bars = 100 μm. (**C**) The rate of colony formation in CFU-F after treatment with Cur for 24 h. The expression of senescence-related genes of *p21* and *p16* (**D**), pro-inflammatory cytokines genes *TNF-α* and *IL-6* (**E**), and stemness markers *Nanog* and *SOX-2* (**F**), as measured by RT-qPCR. The values are the means ± SDs of triplicate experiments. Gene expression was normalized relative to the expression of the *GAPDH*. * *p* < 0.05, ** *p* < 0.01 compared with the control group. ^#^ *p* < 0.05, ^##^ *p* < 0.01 for intragroup comparisons.

The 6th passage cBMSCs were incubated in the absence or presence of Cur at doses of 0.1, 0.5, 1, 5, or 10 μmol/mL (μM) for 12 h, 24 h, 48 h, and 72 h, and their cellular viability was assessed using CCK-8. The results showed that Cur (0.1–10 μM) does not induce a toxic effect in cBMSCs and has palpable effects on increasing the viability of cBMSCs after 24 h of treatment (Figure 3A). To evaluate the effect of Cur on cellular senescence, cBMSCs were exposed to different concentrations of Cur (0.1, 1, and 10 μM) for 24 h. The result showed that the numbers of SA-β-gal-positive cells were significantly decreased in cBMSCs after treatment with Cur (1 μM and 10 μM) compared with the control group (Figure 3B).

CFU-F assays had been used to evaluate the effects of Cur on the self-renewal efficiency of cBMSCs (P6). The dose of 1 μM of Cur was found to be the most effective concentration for improving the efficiency of the CFU-F of cBMSCs. However, the dose of 10 μM Cur had adverse effects on the self-renewal efficiency of cBMSCs (Figure 3C). In addition, the senescence-alleviating effect of Cur was observed at a dose of 1 μM, manifested by the downregulation of *p16*, *IL-6*, and *TNF-α* and upregulation of the expression of *Nanog* and *SOX-2* (Figure 3D–F). Taken together, these results indicate that Cur at a dose of 1 μM can effectively exert cytoprotective effects on senescent cBMSCs.

### 2.4. Cur Treatment Enhanced Autophagic Activity in cBMSCs

**Figure 4:**
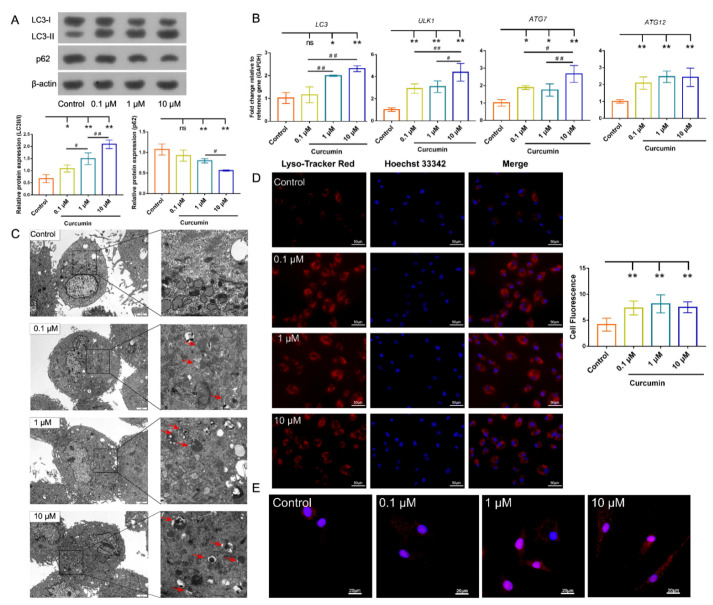
Effects of Cur on the autophagy of cBMSCs (P6). (**A**) Immunoblots showing the protein expression levels of p62, LC3-I, and LC3-II in BMSCs treated or untreated Cur (0.1, 1, and 10 μM), β-actin was used as the loading control. (**B**) The relative expression of *LC3*, *ATG12*, *ATG7*, and *ULK1* in cBMSCs were analyzed using RT-qPCR. (**C**) The ultrastructure of cBMSCs with or without Cur treatment was examined by transmission electron microscopy. The red arrows show autophagosomes and autolysosomes; scale bars = 2 μm. (**D**) cBMSCs were treated or untreated with Cur (0.1, 1, and 10 μM) and then stained with LysoTracker Red DND-99 (60 nM). The relative fluorescence intensity at the intracellular level was quantified using the Image Pro Plus software; scale bars = 50 μm. (**E**) Representative immunofluorescence images show LC3 punctae (red) in BMSCs; scale bars = 20 μm. * *p* < 0.05, ** *p* < 0.01 compared with the control group. ^#^ *p* < 0.05, ^##^ *p* < 0.01 for intragroup comparisons.

Lysosome functional activation plays a critical role in the course of autophagy, and disorders of lysosomal acidification are adverse to the lysosomal degradation function [38]. Therefore, autophagic activity was first detected by LysoTracker staining, which can monitor lysosomal acidification. The result indicated that preconditioning with Cur dose-dependently enhanced the intensity of red fluorescence in cBMSCs, which suggested that lysosome acidification had been increased (Figure 4D).

To further evaluate the effects of Cur on the autophagy of cBMSCs, we made use of the autophagosome markers microtubule-associated protein 1A/1Belight chain 3 (LC3) and p62/SQSTM1, which are pivotal markers in for evaluating autophagic flux [47,48]. Increased autophagic activity was observed in cBMSCs after exposure to Cur, as shown by dose-dependent increase in the conversion of LC3-I to LC3-II, alongside the accelerating p62 degradation (Figure 4A). In addition, the mRNA expression of *ATG7*, *ATG12*, *LC3*, and *ULK1* was upregulated after Cur treatment (Figure 4B). The ultrastructure of cBMSCs was examined using transmission electron microscopy. We then observed an increase in the formation of autophagosomes and autolysosomes in cBMSCs after treatment with Cur at doses of 0.1, 1, and 10 μM for 24 h (Figure 4C). Immunofluorescence staining also showed that the number of characteristic punctate fluorescent dots of LC3 were significantly increased by Cur treatment (Figure 4E). These results indicated that Cur can promote lysosomal acidification and autophagy activation in a dose-dependent manner.

### 2.5. Autophagy Involves in Exerting the Protective Effect of Cur in cBMSC Senescence

**Figure 5:**
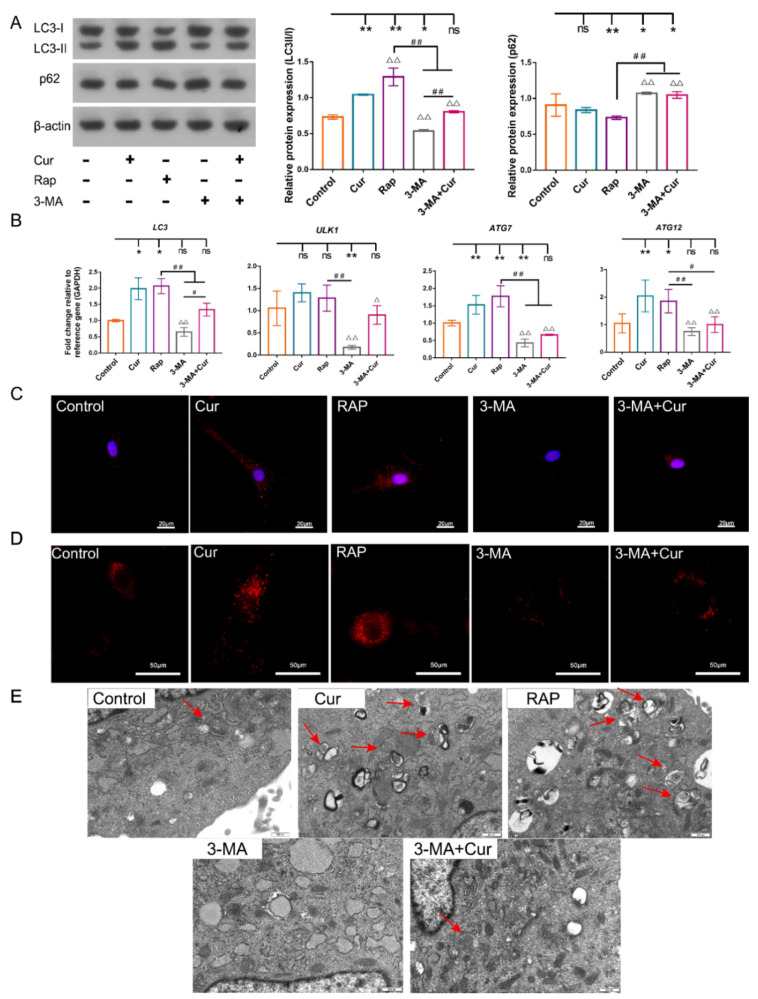
The effects of modulated autophagy through the employment of RAP (100 nM) and 3-MA (5 mM) in cBMSCs (P6). (**A**) Immunoblots showing the protein expression levels of p62, LC3-I, and LC3-II in cBMSCs, β-actin was used as the loading control. (**B**) The relative expressions of *LC3*, *ATG12*, *ATG7*, and *ULK1* in cBMSCs were analyzed using RT-qPCR. (**C**) cBMSCs were treated or untreated with Cur (0.1, 1, and 10 μM) and then stained with LysoTracker Red DND-99 (60 nM); scale bars = 50 μm. (**D**) Representative immunofluorescence images showing LC3 punctae (red) in cBMSCs; scale bars = 50 μm. (**E**) The ultrastructure of cBMSCs was examined with transmission electron microscopy. The red arrows show autophagosomes and autolysosomes; scale bars = 500 nm. * *p* < 0.05, ** *p* < 0.01 compared with the control group; ^Δ^ *p* < 0.05, ^ΔΔ^ *p* < 0.01 compared with the Cur group; ^#^ *p* < 0.05, ^##^ *p* < 0.01 for intragroup comparisons.

To explore the effect of Cur-induced autophagy on cBMSC senescence, the autophagy level was modulated through the employment of RAP (200 nM) or 3-MA (5 mM). 3-MA exerts a significant inhibitory action on autophagic activity, manifested by the significant downregulation of the mRNA expression of microtubule-associated protein 1 light chain 3 (LC3), autophagy-related gene (ATG) 7, ATG12, and unc51-like autophagy-activating kinase-1 (ULK1); the decreased microtubule-associated protein 1 light chain 3 type II/I (LC3-II/I) expression ratio; and the increased expression of p62 compared with the control group (Figure 5A,B). Accordingly, compared with the Cur group, a decrease in the autophagic activity was observed in the 3-MA+Cur group (Figure 5A,B). In contrast, an increase in the autophagic activity was observed in the RAP group, as evidenced by the upregulated mRNA expression of *LC3*, *ATG12*, and *ATG7*; the increased conversion of LC3-I to LC3-II; and the degradation of p62 (Figure 5A,B).

Firstly, the autophagic activity was investigated systematically. The formation of autophagic vacuoles (also termed autophagosomes) was assessed by morphological observation, and the results indicated that there were more autophagic vacuoles and LC3 dots in the Cur group and RAP group compared with the control group, while a reduced number of autophagic vacuoles and fewer LC3 dots were observed in the 3-MA and 3-MA+Cur groups (Figure 5C,E). In addition, LysoTracker staining indicated that lysosomal acidification was enhanced by RAP and Cur in cBMSCs and decreased in the 3-MA and 3-MA+Cur groups (Figure 5D). The results show that Cur and RAP exert a similar positive effect on autophagy activation and that autophagic activity is suppressed by 3-MA. However, the inhibition effects of 3-MA on autophagy can be partially rescued by the employment of Cur.

**Figure 6:**
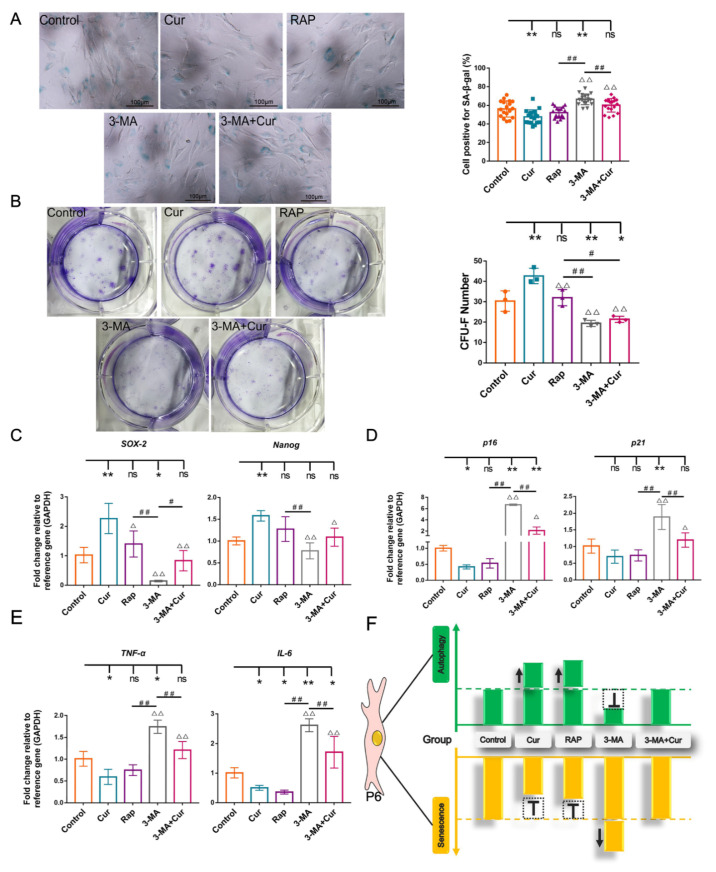
The effects of the modulation of autophagy through the employment of RAP (100 nM) and 3-MA (5 mM) on cBMSC (P6) senescence. (**A**) SA-β-gal expression levels in cBMSCs; scale bars = 100 μm (**B**) The rate of colony formation in CFU-F. The expression of senescence-related genes *p21* and *p16* (**C**), pro-inflammatory cytokine genes *TNF-α* and *IL-6* (**D**), and stemness markers *Nanog* and *SOX-2* (**E**), as measured by RT-qPCR. (**F**) The potential relationship between Cur-induced autophagy and its effects on cBMSC senescence. The values are the means ± SDs of triplicate experiments; * *p* < 0.05, ** *p* < 0.01 compared with the control group; ^Δ^ *p* < 0.05, ^ΔΔ^ *p* < 0.01 compared with the Cur group; ^#^ *p* < 0.05, ^##^ *p* < 0.01 for intragroup comparisons.

To confirm whether autophagy participates in the regulation of cBMSC senescence, we further examined senescence-associated phenotypes after promoting or suppressing autophagy through pharmacological treatment. The results showed that a reduced number of SA-β-gal-positive cells were present in the Cur and RAP groups, while an increased number of SA-β-gal-positive cells were observed in the 3-MA group compared with the control group. It is noteworthy that the number of SA-β-gal-positive cells was significantly increased in the 3-MA+Cur group compared with the Cur group (Figure 6A). The results of a RT-qPCR analysis indicated that treatment with RAP and Cur increased the expression level of *SOX-2* and *Nanog* and decreased the expression level of *IL-6*, *TNF-α*, *p21*, and *p16* compared with the control group. However, the inhibition of autophagy by 3-MA upregulated the expression of *p16*, *p21*, *TNF-α*, and *IL-6* (Figure 6C–E). Furthermore, compared with the control group, the colony-forming efficiency of cBMSCs was increased in the Cur and RAP groups, while the number and the size of CFU-F were significantly decreased in the 3-MA group. Additionally, it was shown that and the enhanced effect of Cur on the colony-forming number of cBMSCs could be abolished through preconditioning with 3-MA (Figure 6B). This evidence indicates that RAP and Cur can ameliorate cBMSC senescence, while 3-MA aggravates cBMSC senescence and attenuates the beneficial effects exerted by Cur.

Taken together, these results suggest that the inhibition of autophagy with 3-MA accelerates cBMSCs senescence, while the activation of autophagy with Cur and RAP alleviates cBMSC senescence (Figure 6F). Notably, the protective effects exerted by Cur were attenuated by pretreatment with 3-MA (Figure 6F), suggesting that Cur-induced autophagy is a potential molecular mechanism for ameliorating cBMSC senescence.

## 3. Discussion

**Figure 7:**
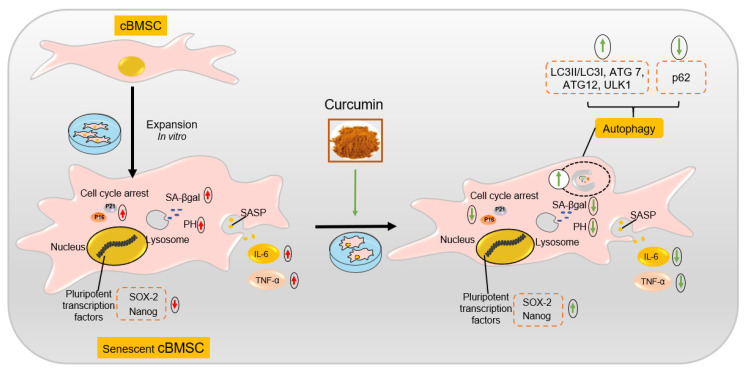
Schematic diagram of the replicative senescence of cBMSCs and the potential mechanisms by which Cur delays cBMSC senescence. cBMSCs (P6) inevitably acquire a senescent phenotype after in vitro expansion, such as a hypertrophic and flat morphology, the activation of cell cycle kinase inhibitors p16 and p21, a decreased expression of pluripotent transcription factors (SOX-2 and Nanog), an enhanced secretion of pro-inflammatory molecules (IL-6 and TNF-α), and an enhanced activity of SA-β-gal and intraluminal pH in lysosomes. Treatment with curcumin can delay the course of cBMSC senescence, while enhancing autophagic activity and promoting lysosomal acidification. All in all, Cur-induced autophagy is a potential molecular mechanism for ameliorating cBMSC senescence.

Organisms continuously repair injured tissues and retard senescence-related processes owing to the distinctively functional characteristics of the MSCs that extensively reside within various tissues and organs [15]. Unfortunately, age and disease are key factors for MSC senescence in vivo, and internal and external differences in cellular environments accelerate MSC senescence in in vitro culture, both of which negatively affect their capacity for immunosuppression, differentiation, and migration, ultimately reducing the efficacy of self-repair and transplantation in MSCs [7,14,49,50,51]. Oja and colleagues indicated that human BMSCs ceased proliferation at the fifth to ninth passage of clinical-grade cultures and exhibited typical senescence phenotypes, such as a hypertrophic and flat morphology, the activation of cell cycle kinase inhibitors p16 and p21, a decreased proliferation rate, and an enhanced activity of SA-β-gal [52]. Evidently, in vitro expansion inevitably engenders premature appearance of senescence in MSCs, which are considered to be an important model system for in vitro cellular aging research [42,44,45]. Our present study found that cBMSCs before the 3rd passage displayed a uniform morphology but had been observed to undergo a series of changes in terms of cellular morphology, physiology and gene expression after the 6th passage (Figure 2 and Figure 7), consistent with premature senescence.

An increasing amount of evidence indicates that pharmacological stimulation is a promising approach for rescuing MSCs from senescence [53,54]. With this in mind, a number of natural and synthetic compounds have been investigated extensively in order to determine their anti-inflammatory, antioxidative and anti-senescence potential in vivo and in vitro [15,55]. As a naturally occurring phenolic compound, Cur has aroused great attention due to its beneficial effects on MSC biology [36,37,56,57]. However, the complex effects of Cur exposure on MSCs should be carefully considered before the implementation of different biomedical research. Yang and colleagues reported that high concentrations of Cur (50 and 100 μM) could induce acute toxic effects in human BMSCs in vitro, while continuous exposure (7 d) to 10 μM of Cur inhibits human BMSC proliferation and induces cell apoptosis [58]. Interestingly, another study indicated that treatment with Cur (≤20 μM) for 5 days ameliorates H_2_O_2_-induced oxidative stress in human ADSCs [56]. Additionally, Cur preconditioning (1 μM and 5 μM) for 24 or 48 h can help to maintain cellular viability and improve the lifespan of rat ADSCs [36,57]. Our results demonstrated that Cur (1 μM and 10 μM) was able to maintain the viability of cBMSCs and alleviate cBMSC senescence after exposure for 24 h, while the colony-forming efficiency of cBMSCs was significantly decreased at a dose of 10 μM (Figure 3). Therefore, the beneficial effects of Cur (10 μM) may be attributed to short-term stimulation, and it can impair the proliferation potential of cBMSCs in the long term.

Recently, the biological activities of Cur have been extensively reported on various in vitro or in vivo models, particularly regarding the modulation of the biological characteristics of MSCs. The anti-aging activity of Cur has been discussed frequently, and Cur has been found to exhibit beneficial effects on aging and age-related diseases at the organismal and cellular levels. However, the mechanism underlying its regulation is complicated due to the different doses and forms of Cur, as well as the mechanism of aging [19]. As a major intracellular mechanism of molecule degradation and organelle turnover, autophagy plays a major role in protecting MSCs against stress conditions and maintaining cellular homeostasis. The modulation of autophagy is considered to be a novel strategy for the amelioration of MSC functions [59]. According to data gathered from substantial studies, the promotion or suppression of autophagy by Cur in various cell models exerts satisfactory cytoprotective effects [38,39,40]. Our data showed that increased autophagic activity was observed after exposure to Cur, as identified by the upregulation of autophagy-related genes (*LC3*, *ULK1*, *Atg7*, and *Atg12*), generation of LC3-II, increase in the number of autophagic vacuoles and acidic vesicular organelles, and a significant decrease in the p62 protein level (Figure 4). Additionally, the lysosome is regarded as an indispensable organelle for autophagy, while the dysregulation of lysosomal pH and alteration of vacuolar H^+^-ATPase (v-ATPase) activity were observed in the process of MSC senescence, thus promoting lysosomal acidification and autophagy and contributing to delaying MSC senescence [60]. Yan and colleagues indicated that Cur can activate the lysosome function of mouse embryonic fibroblasts (MEFs) and induce autophagy, which serves as a crucial survival signal [38]. Similarly, we observed that Cur treatment enhances lysosomal acidification in cBMSCs (Figure 4), suggesting that Cur may be involved in activating lysosome function, which is indispensable for enhancing autophagic activity.

Autophagy is predominantly a cytoprotective mechanism, and an increasing amount of evidence has indicated that the anti-aging properties of natural and synthetic compounds are correlated with autophagy modulation [29,54,61,62]. However, the effects of autophagy modulation on MSC senescence and corresponding mechanisms have not yet been fully evaluated and explored. Initial reports have indicated that autophagy is a predominantly cytoprotective mechanism and that an increased level of autophagy can delay cellular senescence by reducing the accumulation of toxic metabolites and restoring the function of organelles [63]. Interestingly, recent investigations have also shown that increased numbers of autophagic vacuoles and autophagy-related proteins (LC3-II, ATG7, and ATG12) were observed during MSC senescence, while the inhibition of autophagy with bafilomycin A1 and 3-MA was shown to reduce the percentage of SA-β-gal-positive cells and the expression of p16 and p21 [35]. To further elucidate the relationship between Cur-induced autophagy and its effects on cBMSC senescence, autophagy was modulated by pretreatment with rapamycin or 3-MA. Obviously, autophagic activity was found to be attenuated by 3-MA, whereas RAP and Cur (1 μM) were shown to significantly enhance autophagy (Figure 5). Consistent with previous reports [5], our findings also demonstrate that the inhibition of autophagy by 3-MA accelerated cellular senescence in cBMSCs. Almost consistent effects on the activation of autophagy were exerted by Cur (1μM) and RAP, while analogous cytoprotective effects in cBMSCs were displayed. Accordingly, when autophagy was inhibited by 3-MA, the protective effects exerted by Cur were decreased (Figure 6), suggesting that Cur-induced autophagy is a potential molecular mechanism for ameliorating cBMSC senescence (Figure 7).

Under physiological conditions, autophagy occurs at a basal level in all eukaryotic cells to maintain cellular homeostasis. However, various stress conditions can lead to abnormal autophagy, which has an influence on cell fate unless autophagy is restored to an optimal level [41,44,64]. Our evidence confirmed that Cur-induced autophagy exhibits beneficial effects on the regulation of cBMSC senescence. In this scenario, diverse stress-producing stimuli and extracellular settings should be carefully considered before Cur treatment, and it would be interesting to investigate whether Cur-induced autophagy can selectively improve the function of MSCs at a predetermined dose and duration. Additionally, a nanotechnology-based curcumin delivery system has exhibited better aqueous-phase solubility and bioavailability levels [65,66]; it could be a promising tool by which to delay and counteract MSC senescence. The answer to the question of whether autophagy is the primary underlying mechanism in delaying MSC senescence still requires more details that will be provided by future research.

## 4. Materials and Methods

### 4.1. Animals

Bone marrows samples were collected from 6 healthy adult female Chinese rural dogs (12-month-old). All studies were approved by the Faculty Animal Care and Use Committee of Sichuan Agricultural University (approval no.2020-0608) and conducted in accordance with the ethical standards of the animal protection laws of the People’s Republic of China.

### 4.2. Preparation of Curcumin Solution

Cur (HPLC ≥ 98%, CAS number: 458-37-7; Solarbio Science & Technology Co., Ltd., Beijing, China) was dissolved in DMSO to a stock concentration of 20 mmol/L, filtered through a 0.22 μm organic microporous filter membrane and stored at −80 °C. Different Cur solutions were prepared in a medium for in vitro study.

### 4.3. Cell Culture and Expansion

cBMSCs were obtained from bone marrow. The cells were cultured in complete medium consisting of low-glucose Dulbecco’s Modified Eagle Medium (LG-DMEM, Gibco, Grand Island, NY, USA), 10% fetal bovine serum (FBS, TransGen Biotech Co., Ltd., Beijing, China), and 1% penicillin/streptomycin. At an 80–90% confluence, the adherent cells were released with Trypsin Digestion Solution (Beyotime Biotechnology Co., Ltd., Shanghai, China) and further expanded at a ratio of 1:2–1:3 [67].

### 4.4. Cell Growth Curve

To determine the proliferative ability of cBMSCs in passage (P) 3, 6, and 9, the cells were seeded in three 48-well plates (2500 cells/well). After 48 h incubation, the cells were released with Trypsin Digestion Solution and counted with a hemocytometer. The cell counting procedure was repeated every 48 h and sustained for 14 d.

### 4.5. Detection of Immunophenotype of cBMSCs by Flow Cytometry

The 3rd passage cBMSCs were washed with PBS and trypsinized. The cells (3 × 10^5^ cells/mL) were re-suspended in the staining buffer, and the cell suspensions (100 μL) were incubated with FITC, PE, or APC fluorescent-labeled monoclonal antibodies against the surface antigens CD45, CD34, and ITGB1 (eBioscience, San Diego, CA, USA) and unfluorescent-labeled CD31, CD90, and CD105 (Biosynthesis biotechnology Co. Ltd., Beijing, China) for 15 min at 4 °C. The cells were washed with PBS and incubated with FITC-conjugated goat anti-rabbit IgG for 15 min at 4 °C. The surface antigens were detected by flow cytometry (FACS Calibur, Becton Dickinson, San Jose, CA, USA). Data analysis was carried out with the CytExpert software.

### 4.6. In Vitro Differentiation Assay

cBMSCs were plated at a density of 5 × 10^4^ cells/mL in 6-well plates. At 70–80% confluence, the complete medium was replaced with an osteogenic or adipogenic differentiation induction medium and changed every 3 days (Cyagen, Suzhou, China). Calcium deposition was detected by Alizarin Red S staining (Solarbio, Beijing, China) after 3 weeks of osteogenic induction and the lipid droplet accumulation was observed using Oil Red O staining (Solarbio, Beijing, China) after 2 weeks of adipogenic induction.

### 4.7. Effect of Cur on Cellular Viability

The cellular viability of cBMSCs was determined using the CCK-8 kit (Vazyme Biotech Co., Ltd., Nanjing, China). cBMSCs were pre-cultured in a 96-well plate for 24 h. cBMSCs were treated with Cur at different concentrations (0.1, 0.5, 1, 5, and 10 μmol/L) for 12 h, 24 h, 48 h, and 72 h. Cells were treated with 0.1% of DMSO, which was used as a control. After incubating with 10 μL of CCK-8 solution per well for 2 h, the optical density was measured by a microplate reader at 450 nm (Thermo Scientific, Waltham, MA, USA). The relative cell viability was calculated in accordance with the manufacturer’s instructions.

### 4.8. Colony Formation Assay

The self-renewal efficiency of cBMSCs was detected using a colony-forming unit-fibroblast (CFU-F) assay. cBMSCs were seeded (3 × 10^2^ cells/well) in 6-well plates. After two weeks of culture, the cells were fixed with 4% paraformaldehyde for 30 min and observed under an inverted microscope (LX73, Olympus Corporation, Tokyo, Japan) after staining with 1% crystal violet for 10 min. For the CFU-F, more than 50 cells were counted. The CFU-F efficiency was calculated as follows:

CFU-F efficiency = number of CFU-F/number of seed (300 cells) [68].

### 4.9. Beta-Galactosidase Staining Assay

The activity of senescence-associated β-galactosidase (SA-β-gal) in cBMSCs was estimated using the SA-β-gal staining kit (Beyotime Biotechnology Co., Ltd., Shanghai, China) according to the manufacturer’s instructions. After staining, the cells were examined under an inverted microscope. Positively stained cells were counted to assess the cellular senescence.

### 4.10. Reverse Transcription Real-Time Quantitative PCR (RT-qPCR)

The total RNA was extracted from the cell pellets using the Trizol reagent method. cDNA was synthetized using the PrimeScript™ RT reagent kit with the gDNA Eraser (Takara, Shiga, Japan). PCR primers (Table 2) besides *GAPDH* in reference to previous studies [67] were designed using the Primer Express^®^ software (Applied Biosystems, Foster City, CA, USA) based on cDNA sequences. The qPCR was performed using TB^®^ Green PCR Mix (Takara, Shiga, Japan) on the CFX96 Touch Real-time PCR Detection System (Bio-Rad, Richmond, CA, USA). The reaction conditions were as follows: 95 °C for 30 s and then 39 cycles of 95 °C for 5 s and 60 °C for 30 s. A melting curve analysis was performed starting at 95 °C for 10 s, then ranging from 65 to 95 °C, increasing by 0.5 °C every cycle. *GAPDH* was used as an internal control to normalize all the data and the relative expression was calculated through the comparative Cycle Threshold (Ct) method.

### 4.11. Tracking of Lysosomal Using LysoTracker

Lyso-Tracker Red (Beyotime Biotechnology Co., Ltd., Shanghai, China) was used to track lysosomes, which can exhibit an increased fluorescence intensity upon lysosomal acidification. We seeded cBMSCs in 12-well plates and treated them with Cur for 24 h, then the cells were treated with Lyso-Tracker (60 nM) and Hoechst 33,342 (2 μg/mL) for 20 min. The fluorescence was observed using an inverted fluorescence microscope after washing with PBS.

### 4.12. Immunofluorescence

The cBMSCs (2 × 10^4^ cells/slide) were seeded onto slides, and fixed with 4% paraformaldehyde for 30 min. After co-incubation with 0.5% Triton X-100 for 5 min (Solarbio, Beijing, China), the sections were immersed in the blocking solution for 30 min. The enclosed liquid was removed, and cells were incubated with anti-LC3B antibodies (1:1000, Abcam, Cambridge, MA, USA) overnight at 4 °C, then incubated with fluorochrome-conjugated secondary antibodies (Abcam, Cambridge, MA, USA) for 50 min at 37 °C. Finally, the cells were counterstained with DAPI (Beyotime Biotechnology, Shanghai, China) and monitored under a confocal microscope.

### 4.13. Western Blotting Analysis

The cell samples were lysed with the tissue and cell lysate (Solarbio, Beijing, China) containing protease inhibitor after washing with ice-cold PBS. The cell lysates containing 15 μg of protein per sample were loaded into sodium-dodecyl sulfate polyacrylamide (SDS-PA) (Solarbio, Beijing, China) gels and separated by electrophoresis. After transferring the proteins onto a polyvinylidene fluoride (PVDF) membrane, the latter were blocked nonspecifically with 5% nonfat dry milk (Solarbio, Beijing, China) for 1 h at room temperature. The membranes were incubated overnight with the primary antibodies anti-LC3B (1:2000, Abcam, Cambridge, MA, USA), anti-p62/SQSTM1 (1:4000, Novus Biologicals, Littleton, NH, USA), and anti-β-actin (1:1000, Abcam, Cambridge, MA, USA) at 4 °C, and the blots were washed with TBST (Solarbio, Beijing, China) prior to incubating them with secondary antibody (1:2000, Abcam, Cambridge, MA, USA) at 37 °C for 1 h. Subsequently, the membranes were developed by exposure to chemiluminescence reagents (Millipore, Billerica, MA, USA) and visualized with ChemiDocTM Imaging Systems (Tanon-5200, Shanghai, China). The band density was quantified using Image-Pro Plus 6.0 software (Media Cybernetics, Silver Spring, MD, USA) for each group and normalized with β-actin.

### 4.14. Transmission Electron Microscopy (TEM)

The cell pellet was digested and collected into a 1.5 mL centrifuge tube, then fixed with 2.5% glutaraldehyde (Solarbio, Beijing, China) for 2 h at room temperature. The samples were post-fixed with 1% osmium tetroxide for 1 h after washing with PBS, then we increased the dehydration in a stepwise manner in solutions of acetone and embedded them in 812 epoxy resin (Beijing Zhongjingkeyi Technology Co., Ltd., Bejing, China). Subsequently, 50 nm sections were obtained from the ultra-microtome (EM UC7, Leica Microsystems Co., Ltd., Heidelberg, Germany). The sections were stained with uranyl acetate (Zhongjingkeyi, Bejing, China) for 10–15 min and lead citrate (Zhongjingkeyi, Bejing, China) for 2 min. All specimens were viewed on a TEM (JEM-1400PLUS, JEOL, Akishima, Tokyo, Japan).

### 4.15. Statistical Analysis

The results were obtained from three independent experiments and all data were shown as means ± standard deviations (SD). Statistical values were analyzed using the IBM SPSS Statistics 25 and illustrated using GraphPad Prism 9.0 (GraphPad Software, San Diego, CA, USA). Statistically significant differences were determined using a performed using a one-way analysis of variance (ANOVA) and the Student’s *t* test. *p* values < 0.05 were considered to be significant differences.

## 5. Conclusions

Our findings shed light on the relationship between Cur, cBMSC senescence, and autophagy. The data from our study suggests that Cur can alleviate the senescence state of cBMSCs, while activating autophagy and promoting lysosomal acidification. Moreover, further evidence demonstrated that Cur-induced autophagy is a potential mechanism for ameliorating cBMSC senescence. Cur could be a promising activator and conservator for improving the function of MSCs. In our opinion, the positive effects of Cur on aging cannot be neglected. Future studies should focus on the effect of the regulation of Cur on MSC fate to enhance the therapeutic potential of MSCs in various diseases, such as tissue damage and degenerative and inflammatory diseases.

## Figures and Tables

**Table 1:** Flow cytometry analysis of surface antigens in cBMSCs.

Surface Antigens	CD31	CD34	CD45	CD90	CD105	ITGB1
Positive cell rate (%)	6.69 ± 1.62	3.09 ± 0.77	1.50 ± 0.09	98.77 ± 0.80	98.17 ± 0.52	99.63 ± 0.36

**Table 2:** Primers used for real-time quantitative PCR.

Primers	Forward Primer Sequence (5′-3′)	Reverse Primer Sequence (5′-3′)	Product Size (bp)
*GAPDH*	TCCCGCCAACATCAAA	TCACGCCCATCACAAAC	163
*SOX-2*	AACCCCAAGATGCACAACTC	CGGGGCCGGTATTTATAATC	171
*Nanog*	CCTGCATCCTTGCCAATGTC	TCCGGGCTGTCCTGAGTAAG	98
*p16*	CGGAGCCCGATTCAGGTCAT	CACCAGCGTGTCCAGGAAGC	150
*p21*	CATCCCTCATGGCAGCAAG	AGGCAGGGAGACCTTGGACA	208
*IL-6*	TGATGGCTACTGCTTTCCCTACC	CCAGTGCCTCTTTGCTGTCTTC	195
*INF-α*	GCCTCTTCTCCTTCCTCCTC	GCTACTGGCTTGTCACTTGG	169
*LC3*	AGAGCAGCATCCTACCAA	CCATCTTCATCCTTCTCACT	249
*ATG7*	ACGCCAATATCTCCTACTCCAA	CTGCTCTAGTTGCTCCACATC	230
*ATG12*	ATGGCTGAGGAGTCGGAGT	TGGTTCGGGTTCGCTCTAC	241
*ULK1*	TGGAGCAAGAGCACACGGAGA	GGATCTGGTCAATGGCGGTCTG	258

## Data Availability

The datasets generated during and/or analysed during the current study are available from the corresponding author on reasonable request.

## Abbreviations

3-MA	3-Methyladenine
ATG	Autophagy-related gene
cBMSCs	canine bone marrow-derived mesenchymal stem cells
CCK-8	cell counting kit-8
CFU-F	Colony-forming unit-fibroblast
Cur	curcumin
IL	interleukin
LC3	Microtubule-associatedprotein 1 light chain 3
RAP	rapamycin
SA-β-gal	Senescence-associated beta-galactosidase
SASP	senescence-associated secretory phenotype
TEM	transmission electron microscopy
TNF	tumor necrosis Factor
ULK1	unc51-like autophagy-activating kinase-1
